# Association between periodontitis and breast cancer: two-sample Mendelian randomization study

**DOI:** 10.1007/s00784-023-04874-x

**Published:** 2023-02-07

**Authors:** Ming Ding, Zhonghua Zhang, Zhu Chen, Jukun Song, Beichuan Wang, Fuqian Jin

**Affiliations:** 1 School of Stomatology, Zunyi Medical University, Zunyi, China; 2 Department of Endodontics, Guiyang Stomatological Hospital, 253 Jiefang Road, Nanming District, Guiyang, 550005 Guizhou China; 3 Department of Oral and Maxillofacial Surgery, The Affiliated Stomatological Hospital of Guizhou Medical University, Guiyang, China

**Keywords:** Periodontitis, Breast cancer, Mendelian randomization, GWAS

## Abstract

**Objectives:**

The purpose of this study was to investigate whether there is a causal relationship between periodontitis and breast cancer by Mendelian randomization analysis.

**Materials and methods:**

We performed a two-sample bidirectional Mendelian randomization (MR) analysis using publicly released genome-wide association studies (GWAS) statistics. The inverse-variance weighted (IVW) method was used as the primary analysis. We applied complementary methods, including weighted median, weighted mode, simple mode, MR-Egger regression, and MR-pleiotropy residual sum and outlier (MR-PRESSO) to detect and correct for the effect of horizontal pleiotropy.

**Results:**

IVW MR analysis showed no effect of periodontitis on breast cancer (IVW OR=0.99, *P* =0.14). Similarly, no significant causal relationship between breast cancer and periodontitis was found in reverse MR analysis (IVW OR=0.95, *P* =0.83). The results of MR-Egger regression, weighted median, and weighted mode methods were consistent with those of the IVW method. Based on sensitivity analyses, horizontal pleiotropy is unlikely to distort causal estimates.

**Conclusions:**

Although observational studies have reported an association between periodontitis and breast cancer, the results of our MR analysis do not support a causal relationship between periodontitis and breast cancer.

**Clinical relevance:**

Mendelian randomization study can more clearly analyze the causal relationship between periodontitis and breast cancer, in order to provide a certain reference for clinicians and deepen the understanding of the relationship between periodontitis and breast cancer, to explore more possible associations between periodontitis and systemic diseases.

**Supplementary Information:**

The online version contains supplementary material available at 10.1007/s00784-023-04874-x.

## Introduction

Periodontitis is a chronic multifactorial inflammatory disease associated with plaque biofilm, leading to chronic insoluble and destructive inflammatory responses [1]. The overall prevalence of periodontitis is 45 to 50%, and the most severe form of periodontitis affects 11.2% of the world population and is the sixth most common human disease [2]. Studies have shown that periodontal pathogens can be isolated from precancerous lesions and cancerous lesions, demonstrating a pro-cancer microenvironment, and an association between periodontal severity and cancer incidence [3]. Studies have shown that the periodontal pathogens *Porphyromonas gingivalis* and *Fusobacterium nucleatum* are associated with systemic complications [4]. The microbial community context is also relevant to oncopathogenicity, and consortia of *P. gingivalis* and *Fusobacterium nucleatum* are synergistically pathogenic in oral cancer models in vivo [5].

Breast cancer is the most common malignancy in women, accounting for 30% of cases and 15% of deaths in 2021 [6]. Oral microbiome disorders may be associated with breast cancer risk factors [7]. In animal experimental models, *Fusobacterium nucleatum* in the mouth can be transferred to breast tumors through blood circulation, and *Fusobacterium nucleatum* accelerates tumor growth and promotes metastatic progression [8]. Women diagnosed with periodontitis are two to three times more likely to develop breast cancer than women without periodontitis [9]. A meta-analysis showed that periodontal disease significantly increased the risk of breast cancer by 1.22 times [10]. Observational studies showed that periodontal disease increased the susceptibility to breast cancer (RR = 1.18, 95%CI: 1.11–1.26, *I*²= 17.6%), and reliable results were confirmed by sensitivity analysis [11]. The production of IL-1B in periodontal inflammation promotes the expression of CXCL12, and these chemokines promote breast cancer metastasis by recruiting bone marrow-derived suppressor cells (MDSCs) [12].

The causal relation concerning periodontitis and breast cancer risk is limited in terms of evidence, with only observational studies available. Although observational studies have shown that periodontitis increases the risk of breast cancer, there is no evidence for this in Mendelian randomization studies. To overcome the limitations of observational studies, Mendelian randomization (MR) with data from genome-wide association studies (GWAS) could be considered to assess causality in a putative exposition-outcome pathway.

**Fig. 1:**
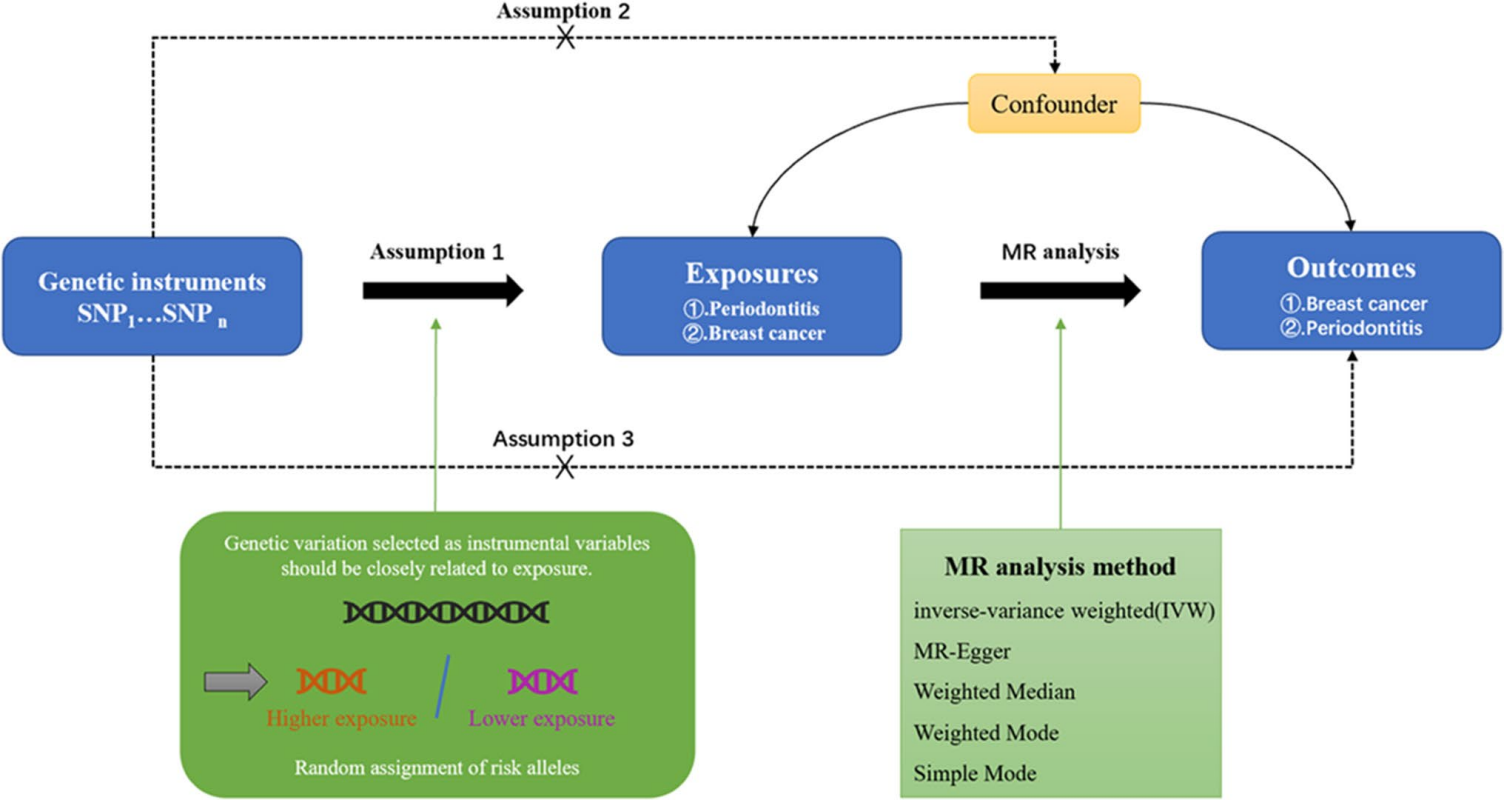
Schematic design of a two-sample Mendelian randomization study of periodontitis and breast cancer. This design assumes a bidirectional association between periodontitis and breast cancer, but no association with confounders. Genetic variants can influence breast cancer through periodontitis and vice versa. SNP stands for single nucleotide diversity

Mendelian randomization (MR) genetic variation was used to determine whether the observed correlation between risk factors and the results is consistent with the causal effect, depends on the nature, random genetic mutations during meiosis to produce random distribution of the individuals of genetic variation at birth were affected by the distribution of natural inheritance a risk factor of genetic variation or not inherit the mutation [13]. MR uses an instrumental variable (IV) to further analyze the causal relationship between samples [14]. These IVs in MR must meet three assumptions: (1) correlation hypothesis: strong correlation with exposure; (2) exclusivity hypothesis: it has nothing to do with the outcome; (3) independence hypothesis: it has nothing to do with confounding factors [15, 16]. Figure 1 shows a schematic of the Mendelian randomization study of periodontitis and breast cancer. At present, there is no association between periodontal disease and breast cancer assessed by MR. We aimed to investigate the relationship between periodontitis and breast cancer, and to further confirm the relationship by conducting a correlation analysis.

## Materials and methods

### Participants and data sources

Periodontitis data was used in this study from the European samples FinnGen project (https://www.finngen.fi/en) [17]. Breast cancer dataset from OpenGWAS database (https://gwas.mrcieu.ac.uk) in the European samples is an open source, open database, through strict quality control [18]. The OpenGWAS Database is a database of publicly available datasets, the University of Helsinki is the organization responsible for the FinnGen Project, and each study included in it was approved by the local institutional review board and ethics committee. The Centers for Disease Control and Prevention (CDC)/American Academy of Periodontology edefinition(AAP), comparable standards assessed by probing depth, or self-reporting was used to categorize periodontitis cases [19]. We conducted a two-way, two-sample MR study to explore the causal association between periodontitis and breast cancer. Three thousand forty-six cases of periodontitis and 195,395 control cases, 76,192 cases of breast cancer, and 63,082 control cases were obtained from GWAS data for the study. The samples were all from people of European ancestry.

### Statistical analysis for Mendelian randomization

Data analysis in this study was performed using R (version4.2.1) through TwoSampleMR(0.5.6) package and MRPRESSO(1.0) [20]. Mendelian randomization (MR) can estimate the causal effect of risk factors on complex diseases using genetic variation as instrumental variable (IV) [21].

The inverse-variance weighted (IVW) method was used as the primary analysis [22]. Multiple complementary MR detection methods were used to accurately examine causal effects and correct for the effect of horizontal pleiotropy, including the weighted median method, weighted mode method, simple mode, MR-Egger regression method, and MR-pleiosis residual and outlier method (MR-PRESSO) [23, 24]. IVs were extracted according to *P* < 5×10−6.

IVW is a valid analysis under the basic premise that all genetic variants are valid instrumental variables and have a strong ability to detect causality [25]. Harmonize removing the SNP for incompatible Alleles and the SNP for being palindromic with Intermediate Allele frequencies. Due to the differences in different experimental conditions, selected populations, and SNPS, the two-sample MR analysis may be heterogeneous, which may bias the estimation of causal effect results. Therefore, heterogeneity test was adopted for the main IVW analysis method and MR-Egger regression in this study, and the *P*-value of the test result was greater than 0.05, so it was considered that there was no heterogeneity among these IVs. One of the assumptions of MR analysis is that instrumental variables can affect outcomes only through exposure, so horizontal pleiotropy should be tested for causal effects between exposure and outcomes [26]. The intercept value in MR-Egger was used to evaluate pleiotropy. If the intercept term was very close to 0, then the MR-Egger regression model was very close to IVW. The lower the possibility of horizontal pleiotropy, the less significant pleiotropy was, indicating that SNP was only associated with exposure, not with other confounding variables [27]. In this study, the *P*-value of the pleiotropy test was used to analyze the existence of pleiotropy. If the *P*-value was greater than 0.05, the possibility of pleiotropy in the causal analysis was considered to be less or non-existent, and its influence could be ignored. We tested the consistency of the results by leave-one-out analysis [28].

## Results

### Causal effects of breast cancer on periodontitis

**Fig. 2:**
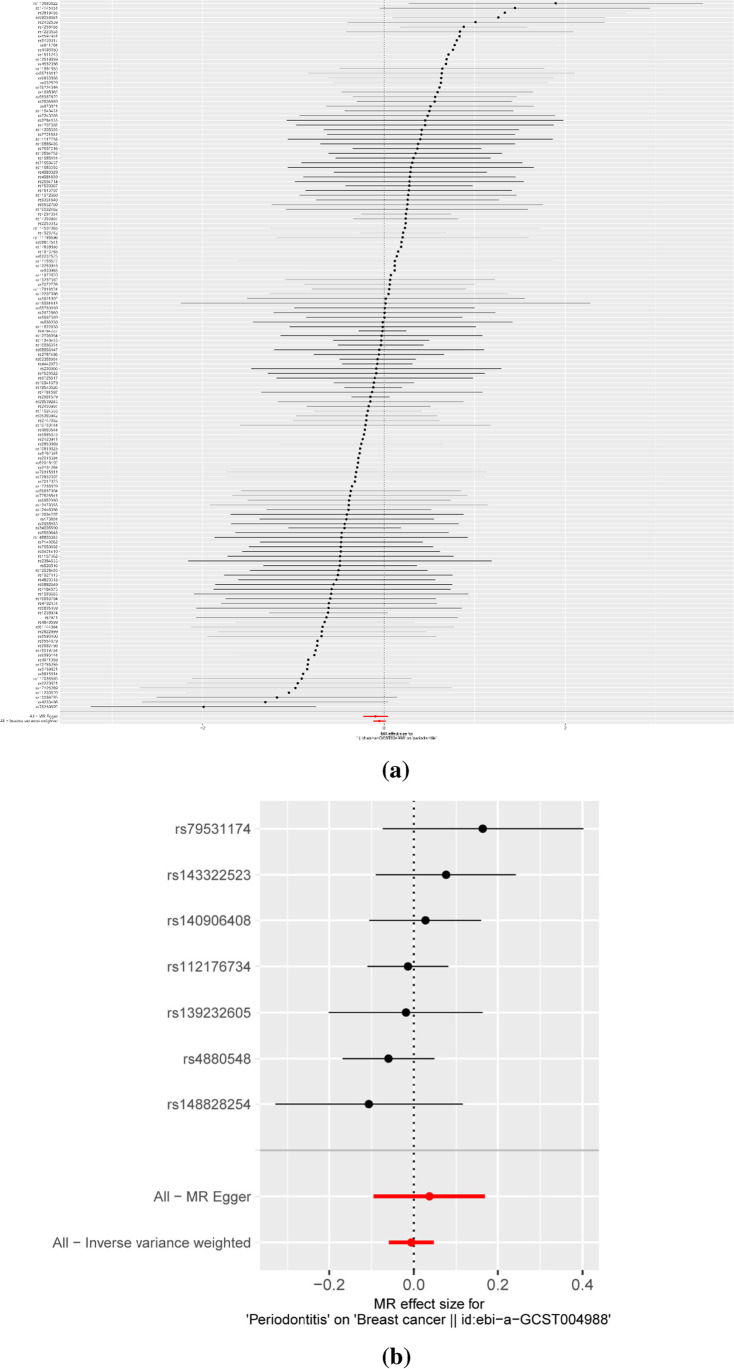
Forest plots of causal effects of breast cancer-associated single nucleotide polymorphisms (SNPs) on periodontitis (**a**) and periodontitis-associated single nucleotide polymorphisms (SNPs) on breast cancer (**b**)

**Fig. 3:**
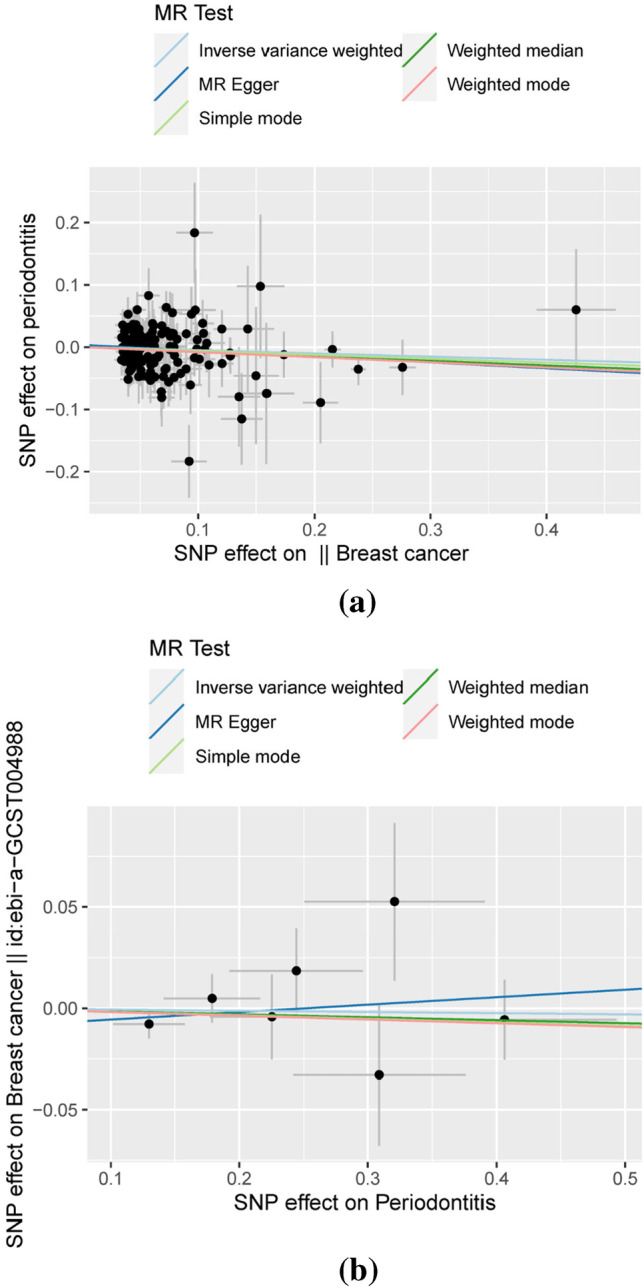
Scatterplot of genetic association between breast cancer and periodontitis. **a** Genetic association of breast cancer with periodontitis and **b** genetic association of periodontitis with breast cancer

In the two-way MR analysis, 171 SNPs were extracted with breast cancer as the exposure and periodontitis as the outcome. The results showed IVW (OR=0.950, 95%CI= 0.888–1.017, *P*=0.1408), MR-egger (OR=0.910, 95%CI=0.794–1.043, *P*=0.1785), and weighted median (OR=0.929, 95%CI=0.833–1.037, *P*=0.1885); breast cancer had no effect on periodontitis (Table 1 and Fig. 3). Cochran’s Q report did not show heterogeneity among these IVs (*P*>0.05). Table 2 shows breast cancer on risk of periodontitis: intercept =0.0035, *P* =0.4774, which suggests that there has no heterogeneity among these IVs. Scatter plots of SNP effect sizes for periodontitis and breast cancer are shown in Fig. 2a and b. The total sample size of periodontitis was 198,441, and the total sample size of breast cancer was 139,274. When there was an effector allele frequency (EAF) value, we calculated *R*² and *F*-statistics by EAF and effect estimate (BETA) to estimate the strength of instrumental variables [29]. The *F*-statistic values are all greater than 10, and the average *F*-statistic value is 237.6 (Table S1). No high-impact points were found in the leave-one-out analysis (Figure S1. Table S2).

**Table 1:** Mendelian randomization estimates for the relationship between genetically instrumented periodontitis and breast cancer

Outcome	Exposure	Method	OR	95%CI	P-value
Periodontitis	Breast cancer	IVW MR-Egger Weighted median Weighted mode Simple mode	0.950 0.910 0.929 0.922 0.940	0.888–1.017 0.794–1.043 0.833–1.037 0.811–1.048 0.743–1.188	0.1408 0.1785 0.1885 0.2137 0.6032
Breast cancer	Periodontitis	IVW MR-Egger Weighted median Weighted mode Simple mode	0.994 1.038 0.985 0.982 0.983	0.943–1.048 0.909–1.184 0.918–1.058 0.908–1.061 0.888–1.088	0.8251 0.6072 0.6848 0.6567 0.7466

**Table 2:** Heterogeneity of Wald ratios and MR-Egger test for directional pleiotropy

Exposure	Heterogeneity				
	Outcome	*Q*	df	*I*²	*P*-value
Breast cancer	Periodontitis	138.699	150	8.14%	0.7359
Periodontitis	Breast cancer	4.931	6	21.68%	0.5527
Exposure	MR-Egger test for directional pleiotropy				
	Outcome	Intercept		SE	*P*-value
Breast cancer	Periodontitis	0.0035		0.0050	0.4774
Periodontitis	Breast cancer	-0.0093		0.0134	0.5179

### Causal effects of periodontitis on breast cancer

**Fig. 4:**
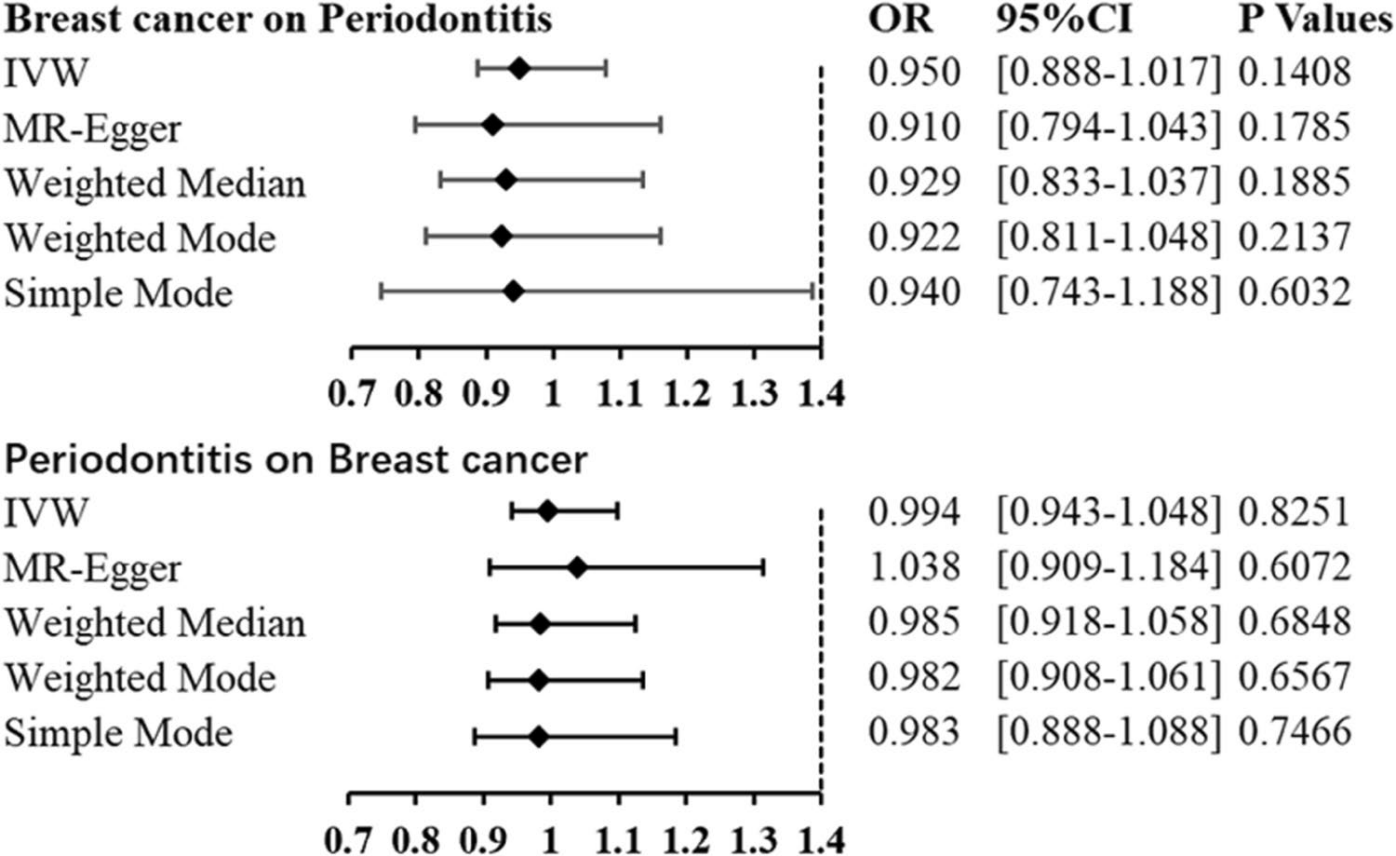
Forest plot. Mendelian randomization estimates for the relationship between genetically instrumented periodontitis and breast cancer, and vice versa. CI, confidence interval; IVW, inverse-variance weighted; OR, odds ratio

Taking periodontitis as exposure and breast cancer as outcome, 7 SNPs were extracted, and the results showed IVW (OR=0.994, 95%CI= 0.943–1.048, *P*=0.8251), MR-egger (OR=1.038, 95%CI=0.909–1.184, *P*=0.6072), and weighted median (OR=0.985, 95%CI=0.918–1.058, *P*=0.6848) which showed that there was no significant correlation between periodontitis and breast cancer (Table 1 and Figs 2b and 3). The *F*-statistic values are all greater than 10, and the average *F*-statistic value is 949.2 (Table S1). The MR-Egger analysis did not show horizontal pleiotropy (periodontitis on risk of breast cancer: intercept =−0.0093, *P* =0.5179 (Table 2 and Fig. 2). The results of leave-one-out analysis showed no obvious abnormalities (Figure S2. Table S2). Our results do not support a bidirectional genetic relationship between periodontitis and breast cancer (Fig. 4).

## Discussion

The results of MR studies do not support evidence of an effect of periodontitis on breast cancer and vice versa. The etiological analysis in MR analysis was used to detect and analyze small and medium effects, and the data were statistically estimated using different MR analysis methods. The results of our MR analysis contradict the results of existing observational studies [9, 11, 30]. However, one study did not observe any association between periodontal disease and breast cancer risk [31]. Although there is some causative association between periodontitis and breast cancer due to their association with microbial and salivary biomarkers, the results of our MR analysis do not support a relationship between the two diseases [7, 32]. It is inferred that either coincidental or confounded by some unknown confounder maybe exist. Moreover, a causal relationship between periodontitis and breast cancer cannot be established in observational studies. Most patients with periodontitis often have a range of systemic problems, and some breast cancer patients have some common diseases, such as diabetes [33–35] and cardiovascular disease [36–38]. Therefore, some inflammatory pathways shared between these diseases may lead to a link between periodontitis and breast cancer.

The same results were obtained by applying complementary MR methods, with OR point estimates slightly above OR below 1 and highly overlapping CIs for the two bidirectional hypotheses: periodontitis affects breast cancer OR breast cancer affects periodontitis. Therefore, it is unlikely that there is a causal link between periodontitis and breast cancer. There are limitations to this MR study. The statistical MR of genetic aggregation limits the scope of analysis, and there are also differences between people. However, as a result of several complementary methods, the effect estimate is close to 1, which is unlikely to be biased in any way. MR analysis usually provides strong evidence in cases where the effect is particularly small or non-existent [39].

## Conclusion

Our findings do not suggest a causal relationship between periodontitis and breast cancer or vice versa. However, careful consideration is needed before generalization of the results, and further studies are needed to verify them, to finally get a more reasonable and powerful result. Clues and evidence from multiple observational and experimental studies can be combined to strengthen causal inference [40].

## Supporting information

ESM 1
