# Supplementary material for: Association between periodontitis and breast cancer: two-sample Mendelian randomization study
Source: Clin Oral Investig. 2023 Feb 7;27(6):2843–9. doi: 10.1007/s00784-023-04874-x (PMC10264523; doi:10.1007/s00784-023-04874-x)

**Table S1. SNPs used as instruments and their association with the exposure and the outcome**

| **SNP** | **CHR** | **EA** | **OA** | **EAF** | **BETA** | **Exposure**  **SE** | **P-value** | **R²** | **F** | **BETA** | **Outcome**  **SE** | **P-value** |
| --- | --- | --- | --- | --- | --- | --- | --- | --- | --- | --- | --- | --- |
| **Breast cancer** | |  |  |  |  |  |  |  |  | **Periodontitis** | |  |
| rs11117758 | 1 | A | G | 0.2086 | -0.0449 | 0.0076 | 3.90E-09 | 0.000666 | 92.7654 | -0.01788 | 0.033537 | 0.593893 |
| rs11205303 | 1 | T | C | 0.3998 | 0.0497 | 0.0064 | 1.14E-14 | 0.001185 | 165.2953 | 0.020527 | 0.027285 | 0.451854 |
| rs11249433 | 1 | T | C | 0.403 | 0.0988 | 0.0065 | 1.76E-52 | 0.004697 | 657.2518 | -0.0031 | 0.026522 | 0.906941 |
| rs11583393 | 1 | C | T | 0.249 | -0.0396 | 0.0072 | 3.61E-08 | 0.000586 | 81.72939 | -0.01169 | 0.027439 | 0.67 |
| rs1707302 | 1 | T | G | 0.6641 | 0.0364 | 0.0066 | 2.95E-08 | 0.000591 | 82.37527 | 0.016414 | 0.026353 | 0.533401 |
| rs17426269 | 1 | A | G | 0.1488 | 0.0487 | 0.0086 | 1.74E-08 | 0.000601 | 83.72348 | -0.04746 | 0.04271 | 0.26643 |
| rs2506889 | 1 | C | A | 0.3185 | -0.0615 | 0.0067 | 2.38E-20 | 0.001642 | 229.0514 | -0.03423 | 0.026911 | 0.20333 |
| rs2784135 | 1 | A | G | 0.4899 | -0.034 | 0.0062 | 4.39E-08 | 0.000578 | 80.51289 | -0.01537 | 0.026484 | 0.561773 |
| rs2992756 | 1 | T | G | 0.5194 | -0.0506 | 0.0063 | 1.60E-15 | 0.001278 | 178.2527 | 0.037205 | 0.025366 | 0.142443 |
| rs35383942 | 1 | A | G | 0.0562 | 0.101 | 0.0139 | 3.79E-13 | 0.001082 | 150.877 | -0.01936 | 0.039833 | 0.626964 |
| rs4233486 | 1 | C | T | 0.6514 | 0.0396 | 0.0069 | 9.09E-09 | 0.000712 | 99.25873 | -0.0519 | 0.027429 | 0.058473 |
| rs4971059 | 1 | T | A | 0.3632 | 0.0424 | 0.0064 | 4.83E-11 | 0.000832 | 115.914 | -0.03533 | 0.025404 | 0.164367 |
| rs59867004 | 1 | C | T | 0.2724 | 0.0446 | 0.007 | 1.40E-10 | 0.000788 | 109.9021 | -0.01635 | 0.027599 | 0.55365 |
| rs666930 | 1 | G | A | 0.5338 | 0.0347 | 0.0063 | 4.69E-08 | 0.000599 | 83.5149 | -0.00046 | 0.025346 | 0.985559 |
| rs72755295 | 1 | G | A | 0.0309 | 0.1376 | 0.0179 | 1.65E-14 | 0.001134 | 158.1068 | -0.11534 | 0.073339 | 0.115788 |
| rs7513707 | 1 | G | A | 0.1661 | 0.0554 | 0.0082 | 1.70E-11 | 0.00085 | 118.5131 | 0.015169 | 0.032203 | 0.637616 |
| rs7529522 | 1 | C | G | 0.2338 | 0.0478 | 0.0075 | 1.73E-10 | 0.000819 | 114.1016 | -0.00409 | 0.02922 | 0.888704 |
| rs79724016 | 1 | T | G | 0.0334 | -0.0975 | 0.0177 | 3.86E-08 | 0.000614 | 85.53876 | -0.05948 | 0.06546 | 0.363504 |
| rs113577745 | 2 | G | A | 0.0992 | 0.064 | 0.0102 | 3.90E-10 | 0.000732 | 102.0262 | -0.0103 | 0.055816 | 0.85355 |
| rs11684853 | 2 | C | G | 0.5606 | -0.0442 | 0.0062 | 1.12E-12 | 0.000962 | 134.1744 | -0.02833 | 0.025423 | 0.265177 |
| rs11693806 | 2 | T | A | 0.7288 | -0.0739 | 0.007 | 3.40E-26 | 0.002159 | 301.314 | -0.00204 | 0.027455 | 0.940654 |
| rs12479355 | 2 | T | C | 0.2088 | -0.0426 | 0.0076 | 2.36E-08 | 0.0006 | 83.55843 | 0.01649 | 0.033349 | 0.620969 |
| rs2016394 | 2 | C | T | 0.4748 | -0.0425 | 0.0062 | 6.23E-12 | 0.000901 | 125.5736 | 0.012011 | 0.025292 | 0.63487 |
| rs34005590 | 2 | G | A | 0.0496 | -0.2051 | 0.0152 | 3.18E-41 | 0.003966 | 554.5479 | 0.089066 | 0.065274 | 0.172409 |
| rs3769821 | 2 | A | G | 0.6683 | -0.0565 | 0.0065 | 3.97E-18 | 0.001415 | 197.3889 | 0.047733 | 0.026088 | 0.067292 |
| **SNP** | **CHR** | **EA** | **OA** | **EAF** | **BETA** | **Exposure**  **SE** | **P-value** | **R²** | **F** | **BETA** | **Outcome**  **SE** | **P-value** |
| rs4442975 | 2 | C | T | 0.5021 | -0.1274 | 0.0061 | 1.14E-95 | 0.008115 | 1139.472 | 0.00953 | 0.025219 | 0.705515 |
| rs4848599 | 2 | C | T | 0.8845 | 0.0933 | 0.01 | 1.58E-20 | 0.001779 | 248.1476 | -0.06091 | 0.046957 | 0.19458 |
| rs6436017 | 2 | T | C | 0.5042 | 0.0399 | 0.0063 | 3.42E-10 | 0.000796 | 110.9417 | 0.031915 | 0.025275 | 0.206685 |
| rs6725517 | 2 | A | G | 0.4088 | -0.0468 | 0.0067 | 2.93E-12 | 0.001059 | 147.6015 | 0.004739 | 0.025905 | 0.854854 |
| rs73949122 | 2 | A | C | 0.1664 | -0.0593 | 0.0084 | 1.85E-12 | 0.000976 | 135.9997 | 0.023141 | 0.03228 | 0.473445 |
| rs76019754 | 2 | C | A | 0.0947 | 0.0745 | 0.0112 | 3.24E-11 | 0.000952 | 132.6668 | -0.05599 | 0.039304 | 0.154276 |
| rs13066793 | 3 | A | G | 0.0925 | -0.0685 | 0.0112 | 1.04E-09 | 0.000788 | 109.8008 | 0.081082 | 0.046245 | 0.07955 |
| rs17838698 | 3 | C | G | 0.2978 | 0.0517 | 0.0067 | 1.58E-14 | 0.001118 | 155.8641 | 0.009711 | 0.02649 | 0.713922 |
| rs3821902 | 3 | C | G | 0.1358 | 0.0623 | 0.0089 | 2.99E-12 | 0.000911 | 126.993 | 0.001231 | 0.048711 | 0.979833 |
| rs56387622 | 3 | T | C | 0.1012 | -0.0942 | 0.0106 | 5.47E-19 | 0.001614 | 225.1857 | -0.05287 | 0.043478 | 0.223953 |
| rs58058861 | 3 | G | A | 0.2172 | 0.0474 | 0.0074 | 1.91E-10 | 0.000764 | 106.4861 | 0.059875 | 0.028406 | 0.035047 |
| rs6787391 | 3 | A | G | 0.3807 | 0.0567 | 0.0064 | 9.07E-19 | 0.001516 | 211.4476 | -0.01525 | 0.025949 | 0.556814 |
| rs6805189 | 3 | G | T | 0.4751 | -0.0339 | 0.0062 | 4.60E-08 | 0.000573 | 79.8737 | 0.020643 | 0.025272 | 0.414019 |
| rs7626742 | 3 | G | C | 0.5327 | 0.1046 | 0.0062 | 1.61E-63 | 0.005447 | 762.7949 | 0.021953 | 0.025337 | 0.38626 |
| rs7650602 | 3 | C | T | 0.4422 | 0.0489 | 0.0063 | 6.22E-15 | 0.00118 | 164.4831 | -0.02327 | 0.025352 | 0.358696 |
| rs9833888 | 3 | T | A | 0.2259 | 0.0457 | 0.0074 | 5.15E-10 | 0.00073 | 101.8021 | 0.028734 | 0.029035 | 0.322353 |
| rs10022462 | 4 | G | A | 0.4379 | 0.0375 | 0.0062 | 1.55E-09 | 0.000692 | 96.48185 | 0.009389 | 0.025566 | 0.713443 |
| rs6815814 | 4 | A | T | 0.2525 | 0.052 | 0.0072 | 6.13E-13 | 0.001021 | 142.3038 | -0.04625 | 0.036763 | 0.20835 |
| rs7697216 | 4 | T | G | 0.8819 | 0.1039 | 0.0098 | 1.61E-26 | 0.002249 | 313.8859 | 0.038095 | 0.037672 | 0.311915 |
| rs77528541 | 4 | T | G | 0.1361 | -0.0583 | 0.0096 | 1.41E-09 | 0.000799 | 111.4037 | 0.021814 | 0.038605 | 0.572028 |
| rs9284657 | 4 | T | C | 0.5005 | 0.0366 | 0.0062 | 3.36E-09 | 0.00067 | 93.34403 | -0.0161 | 0.025301 | 0.524536 |
| rs10054203 | 5 | A | G | 0.4094 | 0.0383 | 0.0065 | 3.21E-09 | 0.000709 | 98.8646 | -0.04138 | 0.025339 | 0.10244 |
| rs10941679 | 5 | A | G | 0.2522 | 0.1278 | 0.0071 | 5.61E-73 | 0.006161 | 863.3161 | -0.01418 | 0.028697 | 0.6212 |
| rs11135046 | 5 | C | A | 0.5529 | -0.0717 | 0.0062 | 4.68E-31 | 0.002542 | 354.8858 | -0.04107 | 0.242926 | 0.865752 |
| rs113993822 | 5 | C | T | 0.0416 | 0.0969 | 0.0157 | 6.61E-10 | 0.000749 | 104.3534 | 0.183525 | 0.080252 | 0.022203 |
| rs12519859 | 5 | G | A | 0.4748 | 0.0365 | 0.0063 | 5.77E-09 | 0.000664 | 92.59843 | 0.025124 | 0.025425 | 0.323085 |
| rs2853669 | 5 | C | T | 0.3061 | -0.0651 | 0.0069 | 4.05E-21 | 0.0018 | 251.1878 | 0.016303 | 0.029823 | 0.584614 |
| rs332529 | 5 | A | C | 0.1605 | -0.0606 | 0.0089 | 9.02E-12 | 0.00099 | 137.9637 | -0.03788 | 0.036562 | 0.300222 |
| rs4081859 | 5 | C | T | 0.7517 | 0.0521 | 0.0073 | 7.08E-13 | 0.001013 | 141.2638 | 0.014783 | 0.031104 | 0.634587 |
| **SNP** | **CHR** | **EA** | **OA** | **EAF** | **BETA** | **Exposure**  **SE** | **P-value** | **R²** | **F** | **BETA** | **Outcome**  **SE** | **P-value** |
| rs4562056 | 5 | C | G | 0.3323 | 0.0416 | 0.0067 | 4.72E-10 | 0.000768 | 107.035 | 0.028355 | 0.028131 | 0.313471 |
| rs4702131 | 5 | A | G | 0.5612 | -0.0424 | 0.0062 | 1.00E-11 | 0.000885 | 123.4225 | 0.025731 | 0.025779 | 0.318209 |
| rs62355901 | 5 | T | G | 0.1602 | 0.1734 | 0.0082 | 3.42E-98 | 0.00809 | 1135.947 | -0.01225 | 0.037159 | 0.741691 |
| rs6554679 | 5 | G | C | 0.7652 | 0.0422 | 0.0075 | 1.97E-08 | 0.00064 | 89.18047 | -0.03085 | 0.028573 | 0.280275 |
| rs6596100 | 5 | A | C | 0.2427 | -0.0439 | 0.0076 | 7.74E-09 | 0.000708 | 98.73437 | 0.03014 | 0.028295 | 0.286793 |
| rs6882649 | 5 | C | T | 0.6651 | 0.0388 | 0.0066 | 3.67E-09 | 0.000671 | 93.46537 | -0.02155 | 0.025885 | 0.405101 |
| rs76651412 | 5 | C | G | 0.0236 | -0.1299 | 0.0232 | 2.23E-08 | 0.000778 | 108.3901 | -0.04681 | 0.097817 | 0.632269 |
| rs7721581 | 5 | C | T | 0.8099 | -0.0605 | 0.0078 | 8.70E-15 | 0.001127 | 157.1476 | -0.02484 | 0.032026 | 0.438003 |
| rs984113 | 5 | C | G | 0.6205 | -0.0369 | 0.0066 | 2.08E-08 | 0.000641 | 89.36731 | 0.01882 | 0.026539 | 0.478228 |
| rs117507065 | 6 | C | G | 0.0543 | -0.0758 | 0.0139 | 4.95E-08 | 0.00059 | 82.23218 | -0.01739 | 0.057602 | 0.762687 |
| rs12207986 | 6 | A | G | 0.5362 | 0.0375 | 0.0062 | 1.45E-09 | 0.000699 | 97.4805 | 0.001825 | 0.025231 | 0.942332 |
| rs2223621 | 6 | T | C | 0.6162 | -0.0408 | 0.0065 | 3.04E-10 | 0.000787 | 109.7445 | 0.038694 | 0.025532 | 0.129649 |
| rs2747652 | 6 | G | A | 0.5263 | 0.0663 | 0.0062 | 1.31E-26 | 0.002192 | 305.9219 | -0.01289 | 0.027176 | 0.635323 |
| rs3819405 | 6 | G | A | 0.3464 | -0.0395 | 0.007 | 1.65E-08 | 0.000707 | 98.46568 | -0.0527 | 0.027031 | 0.051225 |
| rs418053 | 6 | T | A | 0.5685 | -0.0465 | 0.0063 | 1.20E-13 | 0.001061 | 147.9013 | -0.02406 | 0.025523 | 0.345763 |
| rs6569648 | 6 | T | G | 0.7643 | 0.0512 | 0.0073 | 2.98E-12 | 0.000944 | 131.6642 | -0.02384 | 0.030703 | 0.437396 |
| rs76956704 | 6 | C | T | 0.03 | 0.1352 | 0.0182 | 1.27E-13 | 0.001064 | 148.3209 | -0.07979 | 0.079942 | 0.318236 |
| rs9361840 | 6 | A | C | 0.2368 | 0.0541 | 0.0072 | 7.14E-14 | 0.001058 | 147.4919 | 0.014103 | 0.027862 | 0.612735 |
| rs11977670 | 7 | C | A | 0.4315 | 0.0522 | 0.0063 | 1.05E-16 | 0.001337 | 186.4348 | 0.003879 | 0.026521 | 0.883704 |
| rs12706954 | 7 | C | G | 0.3735 | -0.0463 | 0.0066 | 2.43E-12 | 0.001003 | 139.8632 | 0.001308 | 0.026388 | 0.960482 |
| rs17156577 | 7 | G | T | 0.1121 | 0.0578 | 0.0098 | 4.25E-09 | 0.000665 | 92.68471 | 0.006881 | 0.051214 | 0.893118 |
| rs17268829 | 7 | T | C | 0.2804 | 0.0495 | 0.0068 | 4.49E-13 | 0.000989 | 137.8488 | -0.01745 | 0.031235 | 0.576489 |
| rs68056147 | 7 | A | G | 0.299 | 0.0501 | 0.0069 | 5.36E-13 | 0.001052 | 146.6951 | -0.00264 | 0.029716 | 0.92922 |
| rs71559437 | 7 | T | C | 0.122 | -0.0686 | 0.0099 | 5.05E-12 | 0.001008 | 140.5512 | -0.02123 | 0.042576 | 0.618116 |
| rs7781597 | 7 | T | C | 0.3829 | 0.0421 | 0.0064 | 5.12E-11 | 0.000838 | 116.7516 | -0.00558 | 0.02622 | 0.831329 |
| rs7971 | 7 | C | T | 0.3531 | -0.0365 | 0.0065 | 1.93E-08 | 0.000609 | 84.8162 | 0.023115 | 0.02681 | 0.388587 |
| rs10096351 | 8 | T | G | 0.5441 | 0.1055 | 0.0062 | 1.97E-64 | 0.005522 | 773.3067 | -0.00366 | 0.025342 | 0.885019 |
| rs13267382 | 8 | A | G | 0.6493 | -0.0437 | 0.0065 | 1.60E-11 | 0.00087 | 121.2316 | -0.00293 | 0.025744 | 0.909401 |
| rs1511243 | 8 | C | A | 0.8263 | 0.0778 | 0.0083 | 4.34E-21 | 0.001738 | 242.4074 | 0.055149 | 0.031319 | 0.078258 |
| **SNP** | **CHR** | **EA** | **OA** | **EAF** | **BETA** | **Exposure**  **SE** | **P-value** | **R²** | **F** | **BETA** | **Outcome**  **SE** | **P-value** |
| rs2672860 | 8 | A | G | 0.336 | -0.0443 | 0.0066 | 2.32E-11 | 0.000876 | 122.0644 | -0.00029 | 0.02768 | 0.991598 |
| rs4286946 | 8 | T | G | 0.1739 | -0.0799 | 0.0083 | 8.63E-22 | 0.001834 | 255.9275 | 0.002294 | 0.036497 | 0.949876 |
| rs514192 | 8 | A | G | 0.6752 | -0.0383 | 0.0066 | 5.61E-09 | 0.000643 | 89.66425 | -0.04879 | 0.026733 | 0.067993 |
| rs58847541 | 8 | C | T | 0.1471 | 0.0626 | 0.0087 | 5.50E-13 | 0.000983 | 137.082 | 0.011971 | 0.036289 | 0.7415 |
| rs7017073 | 8 | T | A | 0.2115 | 0.0572 | 0.0075 | 2.32E-14 | 0.001091 | 152.1501 | -0.01835 | 0.03195 | 0.56584 |
| rs72658071 | 8 | C | T | 0.0877 | 0.1182 | 0.0107 | 2.58E-28 | 0.002236 | 312.06 | -0.0317 | 0.068907 | 0.645506 |
| rs9693444 | 8 | G | A | 0.6757 | -0.0626 | 0.0066 | 1.60E-21 | 0.001717 | 239.6017 | 0.047986 | 0.026675 | 0.072032 |
| rs970821 | 8 | G | A | 0.401 | 0.0445 | 0.0063 | 2.26E-12 | 0.000951 | 132.6167 | 0.022699 | 0.02599 | 0.382453 |
| rs10760444 | 9 | G | A | 0.5667 | -0.0358 | 0.0062 | 9.06E-09 | 0.000629 | 87.71527 | 0.007313 | 0.025296 | 0.772498 |
| rs10816625 | 9 | C | G | 0.0623 | 0.1094 | 0.0126 | 5.04E-18 | 0.001398 | 195.024 | -0.02874 | 0.051976 | 0.580231 |
| rs10978911 | 9 | T | G | 0.1361 | 0.0839 | 0.0091 | 2.36E-20 | 0.001655 | 230.9185 | -0.00633 | 0.036075 | 0.860819 |
| rs1895062 | 9 | G | A | 0.4036 | -0.049 | 0.0063 | 1.10E-14 | 0.001156 | 161.1673 | -0.02882 | 0.026371 | 0.27439 |
| rs1985742 | 9 | C | G | 0.3516 | 0.0588 | 0.0065 | 8.10E-20 | 0.001576 | 219.9001 | -0.00118 | 0.026947 | 0.965163 |
| rs1999456 | 9 | T | A | 0.5415 | 0.0464 | 0.0062 | 7.77E-14 | 0.001069 | 149.05 | 0.007779 | 0.025348 | 0.758939 |
| rs55760189 | 9 | T | C | 0.1341 | 0.0613 | 0.0095 | 1.38E-10 | 0.000873 | 121.6439 | 0.000545 | 0.031148 | 0.986043 |
| rs630965 | 9 | C | T | 0.6196 | 0.0992 | 0.0064 | 3.21E-54 | 0.004639 | 649.0653 | 0.011654 | 0.026657 | 0.66199 |
| rs10885405 | 10 | G | A | 0.46 | 0.0465 | 0.0062 | 5.44E-14 | 0.001074 | 149.7677 | 0.017389 | 0.02547 | 0.494766 |
| rs11200120 | 10 | A | G | 0.1226 | 0.0682 | 0.0093 | 2.60E-13 | 0.001001 | 139.5036 | -0.07178 | 0.039761 | 0.071012 |
| rs12250948 | 10 | C | T | 0.7844 | -0.0576 | 0.0074 | 1.04E-14 | 0.001122 | 156.4634 | -0.00682 | 0.029199 | 0.815383 |
| rs1268974 | 10 | C | T | 0.6132 | -0.0793 | 0.0063 | 3.71E-36 | 0.002983 | 416.7027 | 0.048746 | 0.02669 | 0.067799 |
| rs1467576 | 10 | T | C | 0.62 | 0.0353 | 0.0064 | 3.05E-08 | 0.000587 | 81.82266 | -0.00663 | 0.026353 | 0.801272 |
| rs1693683 | 10 | A | G | 0.3221 | 0.0367 | 0.0066 | 2.69E-08 | 0.000588 | 81.96677 | -0.02134 | 0.028367 | 0.45188 |
| rs17145151 | 10 | A | C | 0.1203 | 0.0572 | 0.0095 | 1.80E-09 | 0.000693 | 96.51325 | 0.08273 | 0.043536 | 0.057398 |
| rs2420941 | 10 | C | A | 0.4945 | -0.0371 | 0.0063 | 3.57E-09 | 0.000688 | 95.90208 | 0.008712 | 0.026309 | 0.740543 |
| rs2981579 | 10 | A | G | 0.5944 | -0.2376 | 0.0063 | 1.00E-200 | 0.027221 | 3897.168 | 0.035509 | 0.025496 | 0.163705 |
| rs4980029 | 10 | C | G | 0.1638 | 0.0767 | 0.0083 | 2.57E-20 | 0.001612 | 224.8066 | 0.022347 | 0.033078 | 0.499293 |
| rs55716112 | 10 | C | G | 0.0258 | 0.1537 | 0.0202 | 2.76E-14 | 0.001188 | 165.5867 | 0.097526 | 0.114922 | 0.396087 |
| rs61744064 | 10 | T | C | 0.0711 | -0.07 | 0.0122 | 1.01E-08 | 0.000647 | 90.20064 | 0.047304 | 0.051792 | 0.361058 |
| rs7072776 | 10 | G | A | 0.7124 | -0.0618 | 0.0068 | 1.75E-19 | 0.001565 | 218.305 | -0.00383 | 0.029615 | 0.896987 |
| **SNP** | **CHR** | **EA** | **OA** | **EAF** | **BETA** | **Exposure**  **SE** | **P-value** | **R²** | **F** | **BETA** | **Outcome**  **SE** | **P-value** |
| rs72832307 | 10 | A | G | 0.1002 | 0.0611 | 0.0111 | 3.90E-08 | 0.000673 | 93.81718 | -0.01924 | 0.044289 | 0.66403 |
| rs79615811 | 10 | G | T | 0.0256 | 0.1499 | 0.019 | 3.44E-15 | 0.001121 | 156.3009 | -0.04601 | 0.110072 | 0.675922 |
| rs9421410 | 10 | G | C | 0.3269 | -0.0466 | 0.0067 | 2.68E-12 | 0.000956 | 133.2218 | 0.022188 | 0.025928 | 0.39212 |
| rs11822830 | 11 | C | T | 0.5959 | 0.0483 | 0.0063 | 1.98E-14 | 0.001124 | 156.6529 | -0.00073 | 0.025393 | 0.97697 |
| rs148893083 | 11 | T | A | 0.0176 | 0.1589 | 0.0234 | 1.09E-11 | 0.000873 | 121.7088 | -0.0745 | 0.113253 | 0.510654 |
| rs1973765 | 11 | G | A | 0.4001 | -0.0817 | 0.0064 | 9.71E-38 | 0.003204 | 447.6918 | -0.01271 | 0.025669 | 0.620496 |
| rs506516 | 11 | A | T | 0.6604 | 0.0591 | 0.0066 | 3.09E-19 | 0.001567 | 218.5367 | -0.02872 | 0.025563 | 0.261295 |
| rs6597981 | 11 | T | G | 0.5178 | 0.0439 | 0.0062 | 1.35E-12 | 0.000962 | 134.1622 | 0.036465 | 0.025582 | 0.154032 |
| rs78540526 | 11 | T | G | 0.0756 | 0.2758 | 0.0113 | 1.81E-131 | 0.010632 | 1496.601 | -0.03243 | 0.0445 | 0.46616 |
| rs1027113 | 12 | T | C | 0.9138 | 0.0713 | 0.0113 | 2.99E-10 | 0.000801 | 111.6296 | -0.03608 | 0.045823 | 0.431069 |
| rs11049431 | 12 | A | G | 0.2168 | -0.0687 | 0.0077 | 2.97E-19 | 0.001603 | 223.5815 | -0.03423 | 0.032602 | 0.293701 |
| rs111798596 | 12 | A | G | 0.0222 | -0.1428 | 0.0221 | 1.12E-10 | 0.000885 | 123.4065 | -0.02913 | 0.101197 | 0.773467 |
| rs1167362 | 12 | C | A | 0.2598 | 0.0431 | 0.0078 | 3.86E-08 | 0.000714 | 99.57423 | -0.02082 | 0.027387 | 0.447078 |
| rs12422552 | 12 | C | T | 0.2589 | 0.0552 | 0.007 | 3.62E-15 | 0.001169 | 163.038 | 0.012018 | 0.030754 | 0.695956 |
| rs17356907 | 12 | G | A | 0.2985 | -0.0898 | 0.0068 | 1.02E-39 | 0.003377 | 471.941 | -0.02134 | 0.026471 | 0.420186 |
| rs206966 | 12 | C | T | 0.1566 | 0.0487 | 0.0089 | 3.79E-08 | 0.000626 | 87.30704 | -0.00414 | 0.034361 | 0.904048 |
| rs2384633 | 12 | A | C | 0.1919 | -0.0442 | 0.0079 | 2.12E-08 | 0.000606 | 84.43872 | 0.021405 | 0.037741 | 0.570605 |
| rs2464264 | 12 | C | T | 0.4164 | -0.0824 | 0.0063 | 2.13E-39 | 0.0033 | 461.1155 | 0.024347 | 0.025815 | 0.345614 |
| rs7297051 | 12 | C | G | 0.2402 | -0.1204 | 0.0074 | 2.95E-60 | 0.005291 | 740.8378 | -0.02906 | 0.030349 | 0.338323 |
| rs11571833 | 13 | A | G | 0.0081 | 0.2727 | 0.0346 | 3.10E-15 | 0.001195 | 166.6235 | 0.038573 | 0.132879 | 0.771596 |
| rs6562760 | 13 | T | G | 0.766 | 0.0443 | 0.0073 | 1.49E-09 | 0.000704 | 98.05089 | 0.011348 | 0.033864 | 0.737538 |
| rs11624333 | 14 | G | C | 0.2595 | -0.0968 | 0.0071 | 6.50E-42 | 0.003601 | 503.3542 | 0.017317 | 0.029391 | 0.555733 |
| rs117068593 | 14 | A | C | 0.1784 | -0.0589 | 0.0083 | 1.44E-12 | 0.001017 | 141.7822 | 0.053341 | 0.036278 | 0.141467 |
| rs12894297 | 14 | C | T | 0.8316 | -0.0619 | 0.0082 | 6.14E-14 | 0.001073 | 149.6229 | 0.025283 | 0.040493 | 0.532379 |
| rs2253012 | 14 | C | G | 0.4318 | 0.0433 | 0.0065 | 3.13E-11 | 0.00092 | 128.2488 | 0.010279 | 0.025824 | 0.690593 |
| rs4983544 | 14 | C | T | 0.4609 | 0.0353 | 0.0063 | 2.50E-08 | 0.000619 | 86.29552 | -0.00745 | 0.025276 | 0.768174 |
| rs7149262 | 14 | C | G | 0.2113 | -0.0724 | 0.0076 | 2.47E-21 | 0.001747 | 243.7484 | 0.034209 | 0.033218 | 0.303086 |
| rs941764 | 14 | C | G | 0.3429 | 0.0463 | 0.0065 | 8.21E-13 | 0.000966 | 134.6711 | 0.035953 | 0.026512 | 0.175064 |
| rs12594752 | 15 | A | G | 0.1341 | -0.0729 | 0.0092 | 1.86E-15 | 0.001234 | 172.1003 | -0.02528 | 0.035641 | 0.478151 |
| **SNP** | **CHR** | **EA** | **OA** | **EAF** | **BETA** | **Exposure**  **SE** | **P-value** | **R²** | **F** | **BETA** | **Outcome**  **SE** | **P-value** |
| rs12446056 | 16 | T | C | 0.059 | -0.0896 | 0.0137 | 6.44E-11 | 0.000891 | 124.2621 | 0.035014 | 0.041573 | 0.399651 |
| rs2432539 | 16 | G | A | 0.5996 | -0.0349 | 0.0064 | 4.02E-08 | 0.000585 | 81.4994 | -0.03536 | 0.025225 | 0.160949 |
| rs28539243 | 16 | G | A | 0.4878 | 0.0486 | 0.0063 | 9.07E-15 | 0.00118 | 164.5738 | -0.00731 | 0.025406 | 0.773523 |
| rs4496150 | 16 | T | A | 0.2466 | -0.0416 | 0.0072 | 8.09E-09 | 0.000643 | 89.61455 | -0.03152 | 0.02932 | 0.282364 |
| rs4784227 | 16 | T | G | 0.2387 | 0.2153 | 0.0071 | 1.00E-200 | 0.016847 | 2386.542 | -0.00383 | 0.028957 | 0.894841 |
| rs62048402 | 16 | C | T | 0.4156 | -0.0624 | 0.0063 | 3.50E-23 | 0.001891 | 263.9192 | 0.017812 | 0.025481 | 0.484539 |
| rs7184573 | 16 | A | C | 0.3602 | -0.047 | 0.0065 | 5.27E-13 | 0.001018 | 141.9449 | 0.027054 | 0.031298 | 0.387371 |
| rs7500067 | 16 | C | A | 0.2293 | 0.0785 | 0.0073 | 4.06E-27 | 0.002178 | 303.9971 | 0.021841 | 0.028163 | 0.438029 |
| rs2787486 | 17 | C | G | 0.3002 | -0.0758 | 0.0068 | 5.56E-29 | 0.002414 | 337.0284 | 0.004275 | 0.027791 | 0.877756 |
| rs7223535 | 17 | G | T | 0.268 | -0.0439 | 0.007 | 3.53E-10 | 0.000756 | 105.3895 | -0.03662 | 0.028077 | 0.192103 |
| rs117618124 | 18 | T | C | 0.0453 | -0.1074 | 0.0156 | 5.46E-12 | 0.000998 | 139.0916 | -0.00616 | 0.046493 | 0.894668 |
| rs170801 | 18 | A | G | 0.2817 | -0.0591 | 0.007 | 3.17E-17 | 0.001414 | 197.1405 | 0.02445 | 0.029028 | 0.399621 |
| rs527616 | 18 | T | C | 0.6211 | 0.0499 | 0.0064 | 6.70E-15 | 0.001172 | 163.4144 | -0.03635 | 0.025352 | 0.151675 |
| rs7240205 | 18 | T | C | 0.6096 | -0.0365 | 0.0065 | 1.51E-08 | 0.000634 | 88.371 | -0.01752 | 0.026197 | 0.503576 |
| rs9952980 | 18 | C | T | 0.3504 | -0.0478 | 0.0065 | 1.65E-13 | 0.00104 | 145.0146 | 0.018409 | 0.027282 | 0.499827 |
| rs9954058 | 18 | T | G | 0.0721 | -0.0891 | 0.0122 | 2.79E-13 | 0.001062 | 148.0973 | -0.06306 | 0.047954 | 0.188535 |
| rs11672660 | 19 | A | G | 0.213 | 0.0472 | 0.008 | 4.13E-09 | 0.000747 | 104.1014 | 0.012581 | 0.028782 | 0.662042 |
| rs1685191 | 19 | C | A | 0.5647 | -0.0586 | 0.0064 | 3.87E-20 | 0.001688 | 235.5208 | -0.01893 | 0.025516 | 0.458103 |
| rs2594714 | 19 | A | G | 0.2343 | -0.0427 | 0.0075 | 1.08E-08 | 0.000654 | 91.17268 | -0.01197 | 0.027441 | 0.662665 |
| rs2965183 | 19 | T | G | 0.3521 | 0.0445 | 0.0065 | 6.31E-12 | 0.000903 | 125.9448 | -0.01924 | 0.028493 | 0.499458 |
| rs7258465 | 19 | A | G | 0.3436 | -0.0725 | 0.0066 | 2.79E-28 | 0.002371 | 330.9958 | -0.06359 | 0.026023 | 0.014541 |
| rs78269692 | 19 | C | T | 0.0488 | 0.0922 | 0.0154 | 1.92E-09 | 0.000789 | 109.9994 | -0.18345 | 0.058564 | 0.001733 |
| rs16991615 | 20 | T | A | 0.0626 | 0.0758 | 0.0126 | 1.92E-09 | 0.000674 | 93.97762 | 0.001203 | 0.087284 | 0.989003 |
| rs2403907 | 21 | C | T | 0.3159 | -0.0796 | 0.0067 | 1.87E-32 | 0.002739 | 382.4548 | 0.013395 | 0.02764 | 0.627927 |
| rs2822999 | 21 | G | A | 0.173 | 0.0589 | 0.0088 | 2.24E-11 | 0.000993 | 138.3908 | -0.04035 | 0.03435 | 0.240129 |
| rs12628403 | 22 | G | A | 0.096 | 0.0849 | 0.0123 | 5.67E-12 | 0.001251 | 174.4587 | -0.04244 | 0.042329 | 0.316017 |
| rs4820318 | 22 | G | A | 0.3753 | -0.0475 | 0.0064 | 1.03E-13 | 0.001058 | 147.4996 | 0.024847 | 0.026296 | 0.344703 |
| rs5995875 | 22 | C | G | 0.1068 | 0.1206 | 0.0098 | 9.04E-35 | 0.002775 | 387.5388 | -0.02645 | 0.037499 | 0.480576 |
| rs5997389 | 22 | T | G | 0.0903 | 0.0791 | 0.0108 | 2.19E-13 | 0.001028 | 143.311 | 0.000316 | 0.034702 | 0.992735 |
| **SNP** | **CHR** | **EA** | **OA** | **EAF** | **BETA** | **Exposure**  **SE** | **P-value** | **R²** | **F** | **BETA** | **Outcome**  **SE** | **P-value** |
| rs62237573 | 22 | G | A | 0.0092 | 0.4256 | 0.034 | 6.87E-36 | 0.003302 | 461.4317 | 0.059867 | 0.097314 | 0.538429 |
| **Periodontitis** |  |  |  |  |  |  |  |  |  | **Breast cancer** |  |  |
| rs112176734  rs139232605  rs140906408  rs6924687  rs4880548  rs148828254  rs79531174  rs143322523 | 3  3  6  6  10  15  18  19 | C  A  C  T  G  G  T  C | T  G  T  A  A  A  C  A | 0.9729  0.9722  0.914  0.8191  0.2699  0.9875  0.9893  0.9627 | 0.406082  0.225556  0.178776  0.143661  0.129867  0.308971  0.320516  -0.2442 | 0.0873012  0.0485612  0.0376048  0.0305371  0.02809  0.0672038  0.0700698  0.0519663 | 3.30E-06  3.40E-06  1.99E-06  2.55E-06  3.78E-06  4.28E-06  4.78E-06  2.61E-06 | 0.008695508  0.002750041  0.005024503  0.006116222  0.006646804  0.002356745  0.00217491  0.004282734 | 1740.6639  547.2203  1002.0923  1221.1660  1327.8108  468.7749  432.5276  853.5169 | -0.0057  -0.0042  0.0049  -0.0162  -0.0078  -0.0329  0.0526  -0.0186 | 0.0198  0.021  0.0121  0.0083  0.0072  0.035  0.0389  0.0207 | 0.7732  0.8426  0.684101  0.0499804  0.2778  0.3477  0.1766  0.3682 |

**SNP, single nucleoid polymorphism; CHR, chromosome; EA, effect allele. OA, other allele. EAF, effect allele frequency. SE, standard error. R^2^, explained phenotypic variability; F, F statistic; BETA, effect estimate; SE, standard error.**

**Table S2. Leave-one-out analyses**

| **SNP** | **B** | **SE** | **P-value** |
| --- | --- | --- | --- |
| **Breast Cancer - Periodontitis** | | | |
| rs10022462 | -0.05179 | 0.034673 | 0.135288 |
| rs10096351 | -0.05135 | 0.034994 | 0.142248 |
| rs1027113 | -0.04968 | 0.034679 | 0.151959 |
| rs10760444 | -0.05064 | 0.03467 | 0.144132 |
| rs10816625 | -0.04988 | 0.034721 | 0.15086 |
| rs10885405 | -0.05271 | 0.034698 | 0.128714 |
| rs10941679 | -0.04955 | 0.035047 | 0.157451 |
| rs11049431 | -0.05395 | 0.034721 | 0.120243 |
| rs11117758 | -0.05197 | 0.034665 | 0.13379 |
| rs111798596 | -0.05162 | 0.03467 | 0.136531 |
| rs11200120 | -0.04746 | 0.034689 | 0.171258 |
| rs11205303 | -0.05286 | 0.034697 | 0.127639 |
| rs11249433 | -0.05134 | 0.03492 | 0.141509 |
| rs113993822 | -0.05441 | 0.034658 | 0.116419 |
| rs11583393 | -0.05187 | 0.034671 | 0.134613 |
| rs11624333 | -0.04932 | 0.034856 | 0.157061 |
| rs11672660 | -0.05203 | 0.034684 | 0.133554 |
| rs1167362 | -0.04972 | 0.03468 | 0.151661 |
| rs11684853 | -0.05352 | 0.034691 | 0.122862 |
| rs117068593 | -0.0483 | 0.034683 | 0.163763 |
| rs117507065 | -0.05159 | 0.034664 | 0.136673 |
| rs117618124 | -0.0517 | 0.034739 | 0.136658 |
| rs11822830 | -0.05116 | 0.034704 | 0.140404 |
| rs11977670 | -0.05159 | 0.034709 | 0.137169 |
| rs12207986 | -0.05127 | 0.034674 | 0.139228 |
| rs12250948 | -0.0518 | 0.034709 | 0.135588 |
| rs12446056 | -0.0491 | 0.034725 | 0.15734 |
| rs12479355 | -0.05035 | 0.034662 | 0.146352 |
| rs12519859 | -0.05284 | 0.034671 | 0.12751 |
| rs12594752 | -0.05301 | 0.034715 | 0.126746 |
| rs12628403 | -0.04883 | 0.034712 | 0.159503 |
| rs1268974 | -0.04498 | 0.034813 | 0.196376 |
| rs12706954 | -0.05109 | 0.034692 | 0.140832 |
| rs12894297 | -0.05 | 0.034677 | 0.149314 |
| rs13066793 | -0.04802 | 0.034674 | 0.166089 |
| rs13267382 | -0.05142 | 0.034688 | 0.138276 |
| rs148893083 | -0.05002 | 0.034669 | 0.149096 |
| rs1511243 | -0.05667 | 0.034757 | 0.102994 |
| rs1685191 | -0.05339 | 0.034738 | 0.124327 |
| rs1693683 | -0.04994 | 0.034663 | 0.14966 |
| **SNP** | **B** | **SE** | **P-value** |
| **Breast Cancer - Periodontitis** | | | |
| rs16991615 | -0.05107 | 0.034644 | 0.140462 |
| rs1707302 | -0.05216 | 0.034668 | 0.132452 |
| rs170801 | -0.0492 | 0.034715 | 0.156444 |
| rs17145151 | -0.05411 | 0.034664 | 0.118509 |
| rs17156577 | -0.05127 | 0.034655 | 0.139041 |
| rs17268829 | -0.0501 | 0.03468 | 0.148594 |
| rs17356907 | -0.05505 | 0.03487 | 0.114425 |
| rs17426269 | -0.04956 | 0.034655 | 0.152652 |
| rs17838698 | -0.0521 | 0.034708 | 0.133304 |
| rs1895062 | -0.05366 | 0.0347 | 0.121978 |
| rs1973765 | -0.05355 | 0.03484 | 0.124311 |
| rs2016394 | -0.05022 | 0.034687 | 0.14767 |
| rs206966 | -0.05092 | 0.03467 | 0.141875 |
| rs2223621 | -0.04825 | 0.034681 | 0.164146 |
| rs2253012 | -0.05198 | 0.034687 | 0.13397 |
| rs2384633 | -0.05029 | 0.034657 | 0.146729 |
| rs2403907 | -0.04983 | 0.034802 | 0.152202 |
| rs2420941 | -0.05057 | 0.03467 | 0.144686 |
| rs2432539 | -0.05346 | 0.034668 | 0.123092 |
| rs2464264 | -0.04798 | 0.034842 | 0.168455 |
| rs2506889 | -0.05484 | 0.034737 | 0.114426 |
| rs2594714 | -0.05197 | 0.034679 | 0.133958 |
| rs2672860 | -0.05118 | 0.034681 | 0.139986 |
| rs2747652 | -0.04998 | 0.034752 | 0.150415 |
| rs2784135 | -0.052 | 0.034662 | 0.133545 |
| rs2787486 | -0.05096 | 0.034784 | 0.142918 |
| rs2822999 | -0.04876 | 0.034689 | 0.159807 |
| rs2853669 | -0.04986 | 0.034728 | 0.151066 |
| rs28539243 | -0.05057 | 0.034704 | 0.145081 |
| rs2965183 | -0.04989 | 0.034679 | 0.150272 |
| rs2981579 | -0.03956 | 0.036585 | 0.279513 |
| rs2992756 | -0.04773 | 0.034711 | 0.169145 |
| rs332529 | -0.05324 | 0.034685 | 0.124789 |
| rs34005590 | -0.04642 | 0.034835 | 0.182718 |
| rs35383942 | -0.04991 | 0.034762 | 0.151041 |
| rs3769821 | -0.04652 | 0.034726 | 0.180395 |
| rs3819405 | -0.05456 | 0.034673 | 0.115567 |
| rs3821902 | -0.05115 | 0.034662 | 0.140063 |
| rs4081859 | -0.05214 | 0.034687 | 0.132816 |
| rs4233486 | -0.04785 | 0.034672 | 0.16755 |
| rs4442975 | -0.05026 | 0.035171 | 0.153028 |
| **SNP** | **B** | **SE** | **P-value** |
| **Breast Cancer - Periodontitis** | | | |
| rs4496150 | -0.05296 | 0.03467 | 0.126599 |
| rs4562056 | -0.05293 | 0.034674 | 0.126858 |
| rs4702131 | -0.0492 | 0.034684 | 0.156061 |
| rs4784227 | -0.05337 | 0.035836 | 0.136445 |
| rs4820318 | -0.04915 | 0.034696 | 0.156583 |
| rs4848599 | -0.04814 | 0.03471 | 0.165433 |
| rs4971059 | -0.04839 | 0.034686 | 0.163029 |
| rs4980029 | -0.05323 | 0.03474 | 0.125477 |
| rs4983544 | -0.05063 | 0.034669 | 0.144168 |
| rs506516 | -0.0482 | 0.03474 | 0.165286 |
| rs55716112 | -0.05248 | 0.034665 | 0.130047 |
| rs55760189 | -0.05129 | 0.034709 | 0.13951 |
| rs56387622 | -0.05447 | 0.034726 | 0.11673 |
| rs58058861 | -0.05541 | 0.034686 | 0.110165 |
| rs58847541 | -0.05187 | 0.03469 | 0.134819 |
| rs59867004 | -0.05002 | 0.034683 | 0.149273 |
| rs5995875 | -0.04889 | 0.034845 | 0.160568 |
| rs5997389 | -0.05135 | 0.034737 | 0.139322 |
| rs61744064 | -0.04964 | 0.034666 | 0.152198 |
| rs62048402 | -0.04931 | 0.034753 | 0.15595 |
| rs62237573 | -0.05551 | 0.035032 | 0.113095 |
| rs62355901 | -0.05048 | 0.035089 | 0.150254 |
| rs630965 | -0.05385 | 0.034919 | 0.123029 |
| rs6436017 | -0.05356 | 0.03468 | 0.122509 |
| rs6554679 | -0.04922 | 0.034674 | 0.155716 |
| rs6562760 | -0.05164 | 0.034664 | 0.136303 |
| rs6569648 | -0.04962 | 0.034686 | 0.152564 |
| rs6596100 | -0.04917 | 0.034678 | 0.156246 |
| rs6597981 | -0.05413 | 0.034689 | 0.118653 |
| rs666930 | -0.05109 | 0.034667 | 0.140538 |
| rs6725517 | -0.05081 | 0.034696 | 0.143081 |
| rs6787391 | -0.04975 | 0.034728 | 0.151961 |
| rs6805189 | -0.0498 | 0.034666 | 0.150832 |
| rs68056147 | -0.051 | 0.034687 | 0.141477 |
| rs6815814 | -0.04899 | 0.03467 | 0.157637 |
| rs6882649 | -0.04964 | 0.034675 | 0.152228 |
| rs7017073 | -0.04997 | 0.034695 | 0.149821 |
| rs7072776 | -0.0516 | 0.034719 | 0.137218 |
| rs7149262 | -0.04859 | 0.034727 | 0.161736 |
| rs71559437 | -0.05213 | 0.034682 | 0.132801 |
| rs7184573 | -0.04958 | 0.034675 | 0.152724 |
| **SNP** | **B** | **SE** | **P-value** |
| **Breast Cancer - Periodontitis** | | | |
| rs7223535 | -0.05361 | 0.034679 | 0.122133 |
| rs7240205 | -0.05225 | 0.034669 | 0.131806 |
| rs7258465 | -0.05973 | 0.03479 | 0.086026 |
| rs72755295 | -0.04767 | 0.034701 | 0.169531 |
| rs72832307 | -0.0504 | 0.034668 | 0.145974 |
| rs7297051 | -0.05663 | 0.03496 | 0.105257 |
| rs7500067 | -0.0541 | 0.034791 | 0.119921 |
| rs7513707 | -0.05216 | 0.03469 | 0.132653 |
| rs7529522 | -0.0509 | 0.034684 | 0.142262 |
| rs76019754 | -0.04798 | 0.034703 | 0.166829 |
| rs7626742 | -0.05645 | 0.034988 | 0.106653 |
| rs7650602 | -0.0491 | 0.034706 | 0.157114 |
| rs76956704 | -0.04915 | 0.034688 | 0.156492 |
| rs7697216 | -0.05485 | 0.034787 | 0.114848 |
| rs7721581 | -0.05299 | 0.034703 | 0.126762 |
| rs77528541 | -0.05012 | 0.034676 | 0.148341 |
| rs7781597 | -0.05075 | 0.034682 | 0.143355 |
| rs78269692 | -0.04523 | 0.03468 | 0.192181 |
| rs78540526 | -0.04779 | 0.035454 | 0.177658 |
| rs79615811 | -0.05044 | 0.034667 | 0.145698 |
| rs7971 | -0.04971 | 0.034667 | 0.15159 |
| rs79724016 | -0.05277 | 0.034674 | 0.128038 |
| rs9361840 | -0.05242 | 0.034707 | 0.130931 |
| rs941764 | -0.05404 | 0.034692 | 0.119268 |
| rs9421410 | -0.04935 | 0.034695 | 0.154885 |
| rs9693444 | -0.04625 | 0.034743 | 0.183122 |
| rs970821 | -0.05299 | 0.034689 | 0.126646 |
| rs9833888 | -0.05303 | 0.03468 | 0.126214 |
| rs9952980 | -0.04977 | 0.034692 | 0.151374 |
| All | -0.05101 | 0.034628 | 0.140754 |
| **Periodontitis - Breast Cancer** | | | |
| rs112176734 | -0.0024 | 0.032547 | 0.941322 |
| rs139232605 | -0.00482 | 0.028293 | 0.86481 |
| rs140906408 | -0.01234 | 0.029536 | 0.67602 |
| rs143322523 | -0.01531 | 0.028566 | 0.591906 |
| rs148828254 | 0.000103 | 0.027878 | 0.997056 |
| rs4880548 | 0.010945 | 0.031019 | 0.724217 |
| rs79531174 | -0.01489 | 0.02777 | 0.59185 |
| All | -0.00598 | 0.02707 | 0.825053 |

**SNP, single nucleoid polymorphism; B, intercept; SE, standard error; Breast Cancer – Periodontitis, exposure:breast cancer, outcome: periodontitis; Periodontitis - Breast Cancer, exposure: periodontitis, outcome: breast cancer.**

**Figure S1. MR leave-one-out sensitivity analysis for breast cancer on periodontitis.**

**
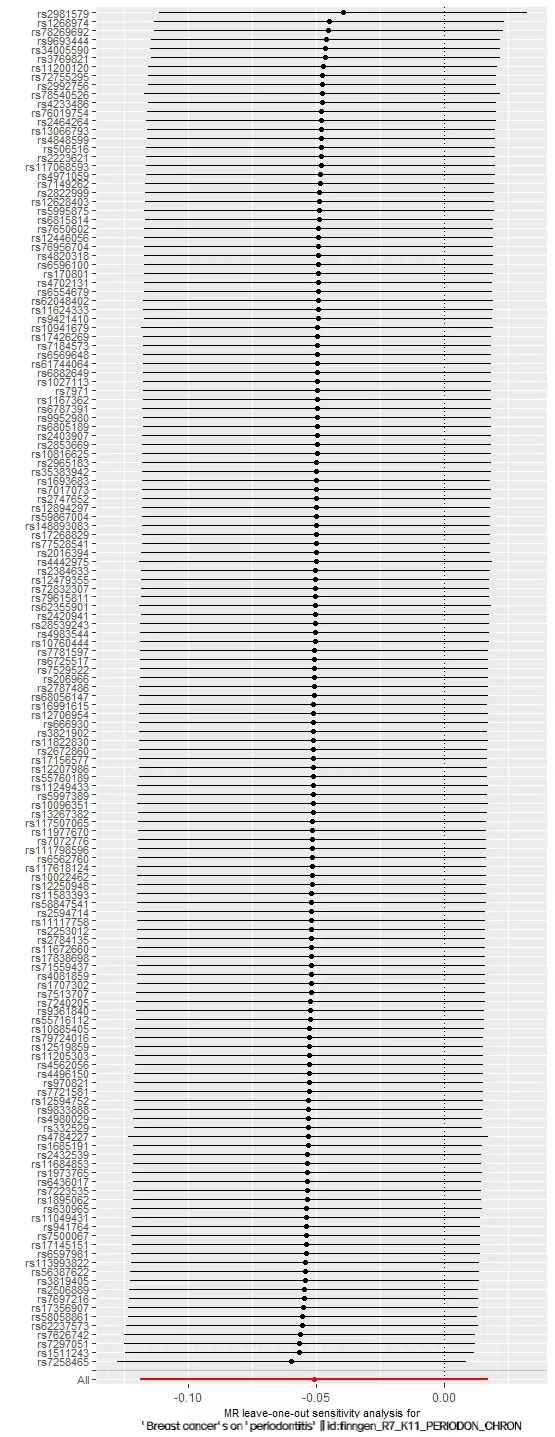
**

**Figure S2. MR leave-one-out sensitivity analysis for periodontitis on breast cancer.**


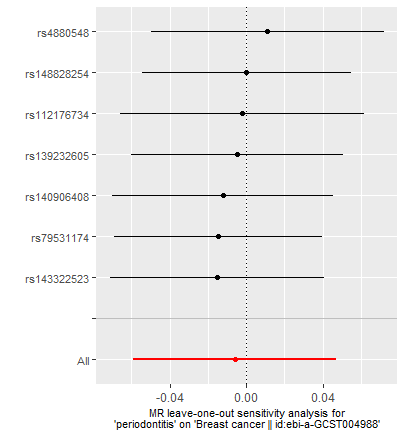

Supplement: Supplementary file 1 — ESM 1 [file 784_2023_4874_MOESM1_ESM.docx]
